# Supplementary material for: Association between Branched-Chain Amino Acid Intake and Physical Function among Chinese Community-Dwelling Elderly Residents
Source: Nutrients. 2022 Oct 18;14(20):4367. doi: 10.3390/nu14204367 (PMC9611152; doi:10.3390/nu14204367)
Supplement: Supplementary file 1 [file nutrients-14-04367-s001.zip › nutrients-1894108-supplementary.pdf]

nutrients

Supplementary Materials

Association between Branched-Chain Amino Acid Intake and Physical Function among Chinese Community-Dwelling Elderly Residents

Minqi Liao [1,2,3,4,5,†], Yingjun Mu [1,2,4,5,†], Xin Su [2,4,5], Lu Zheng [2,4,5], Shiwen Zhang [2,4,5], Hongen Chen [1], Shan Xu [1], Junrong Ma [2,4,5], Ruiqing Ouyang [2,4,5], Wanlin Li [2,4,5], Chen Cheng [2,4,5], Jun Cai [2,4,5], Yuming Chen [6], Changyi Wang [1,*] and Fangfang Zeng [2,4,5,*]

Supplementary materials

Table legends:

- Table S1. Leading seven nutrients of study participants by quartiles of isoleucine, leucine, valine intake (n = 4336)
- Table S2. Covariate-adjusted mean changes in four physical performance indicators by quartiles of total BCAAs intakes in sup-population excluding those with coronary heart disease (n = 3753)
- Table S3. Covariate-adjusted mean changes in four physical performance indicators by quartiles of total BCAAs intakes in sup-population excluding those with myocardial infarction (n = 4273)
- Table S4. Covariate-adjusted mean changes in four physical performance indicators by quartiles of total BCAAs intakes in sup-population excluding those with stroke (n = 4166)
- Table S5. Covariate-adjusted mean changes in four physical performance indicators by quartiles of total BCAAs intakes in sup-population excluding those with angina pectoris (n = 4256)

Table S1. Leading seven nutrients of study participants by quartiles of isoleucine, leucine, valine intake (*n* = 4336).

	Isoleucine				Leucine				Valine			
	**Q1[a]**	**Q4 [a]**	***P*-value [b]**	***P*-trend [c]**	**Q1[a]**	**Q4 [a]**	***P*-value [b]**	***P*-trend [c]**	**Q1[a]**	**Q4 [a]**	***P*-value [b]**	***P*-trend [c]**
Energy intake (kcal/d)	1412.85 ± 521.31	1418.22 ± 486.06	0.852	0.786	1415.680 ± 521.16	1414.78 ± 487.52	0.872	0.896	1413.75 ± 519.99	1421.41 ± 491.72	0.911	0.607
Fat (g/day)	128.95 ± 91.81	120.93 ± 124.75	<0.001	0.020	129.15 ± 91.82	120.41 ± 123.66	0.001	0.027	129.70 ± 91.77	120.91 ± 123.68	<0.001	0.024
Protein (g/day)	53.61 ± 13.82	53.76 ± 14.9	0.744	0.968	54.11 ± 17.39	53.96 ± 15.42	0.923	0.888	54.01 ± 17.21	54.05 ± 15.43	0.952	0.961
Carbohydrate (g/day)	101.13 ± 90.16	114.68 ± 91.51	<0.001	<0.001	100.51 ± 89.92	114.06 ± 91.48	<0.001	<0.001	99.94 ± 90.04	113.54 ± 91.5	<0.001	<0.001
Dietary soluble fiber (g/day)	6.44 ± 8.41	7.03 ± 8.45	0.138	0.031	6.46 ± 8.44	6.95 ± 8.42	0.317	0.080	6.44 ± 8.46	6.95 ± 8.43	0.243	0.063
Vitamin D (µg/day)	144.95 ± 167.94	130.83 ± 236.55	0.004	0.026	145.05 ± 167.98	127.09 ± 200.60	0.020	0.018	146.00 ± 168.71	128.05 ± 200.18	0.011	0.020
Folate (µg/day)	170.22 ± 127.72	187.33 ± 130.41	0.002	<0.001	171.96 ± 128.07	185.16 ± 128.84	0.009	0.006	171.34 ± 128.63	185.78 ± 129.64	0.006	0.003

Abbreviations: Q, quartile.

Note: Data are presented as mean ± standard deviation (SD);

[a] Cutoff values of BCAA quartiles are as follows:

Isoleucine: Q1:< 659.69 mg/d, Q2: 659.69~ 850.09 mg/d, Q3: 850.09~934.89 mg/d, Q4: ≥934.89 mg/d;

Leucine: Q1:< 1268.53 mg/d, Q2: 1268.53~1630.08 mg/d, Q3: 1630.08~1781.98 mg/d, Q4: ≥1781.98 mg/d;

Valine: Q1:< 876.02 mg/d, Q2: 876.02~1158.71mg/d, Q3: 1158.71~1257.01 mg/d, Q4: ≥1257.01 mg/d;

[b] *P*-value was calculated using the ANCOVA analysis for difference across quartiles of each type of BCAAs;

[c] *P*-trend was determined using a test for linear trend across quartiles of BCAAs.

Table S2. Covariate-adjusted mean changes in four physical performance indicators by quartiles of total BCAAs intakes in sup-population excluding those with coronary heart disease (n = 3753).

	Quartiles of BCAAs intake [a]									
	Q1		Q2		Q3		Q4		MD	*P*-trend
	Mean	SE	Mean	SE	Mean	SE	Mean	SE		
Handgrip strength										
Crude	21.5	0.27	22.7	0.27	23.7	0.27	24.4	0.27	2.90	<0.001
Model 1	22.7	0.23	23.6	0.23	24.2	0.23	24.4	0.23	1.66	<0.001
Model 2	22.7	0.31	23.6	0.29	24.2	0.30	24.5	0.30	1.70	<0.001
Model 3	22.7	0.31	23.6	0.29	24.2	0.30	24.4	0.30	1.71	<0.001
4-meter usual walking speed										
Crude	4.0	0.09	4.0	0.09	4.1	0.09	3.7	0.09	−0.28	0.025
Model 1	4.0	0.09	4.0	0.09	4.1	0.09	3.8	0.09	0.24	0.071
Model 2	4.1	0.13	4.1	0.13	4.2	0.13	3.9	0.13	0.22	0.149
Model 3	4.1	0.13	4.1	0.13	4.2	0.13	3.8	0.13	0.22	0.135
4-meter fast walking speed										
Crude	3.1	0.03	3.0	0.03	2.9	0.03	2.8	0.03	0.28	<0.001
Model 1	3.0	0.03	3.0	0.03	2.9	0.03	2.8	0.03	0.24	<0.001
Model 2	3.0	0.04	3.0	0.04	2.9	0.04	2.8	0.04	0.24	<0.001
Model 3	3.0	0.04	3.0	0.04	2.9	0.04	2.8	0.04	0.25	<0.001
Repeated chair rises										
Crude	11.7	0.13	11.4	0.13	11.0	0.13	10.7	0.13	0.99	<0.001
Model 1	11.7	0.12	11.3	0.12	11.0	0.12	10.8	0.12	0.85	<0.001
Model 2	11.9	0.16	11.3	0.16	11.1	0.16	10.9	0.16	0.90	<0.001
Model 3	11.9	0.16	11.3	0.16	11.1	0.16	10.9	0.16	0.92	<0.001

Abbreviations: BCAAs, Branched-chain amino acids; Q, quartile; SE, standard error; MD, mean difference.

Note:

[a] Cutoff values of BCAA quartiles are as follows: Q1:<131,71.538 mg/d, Q2:131,71.538~18194.861 mg/d, Q3:181,94.861~244,28.666 mg/d, Q4: ≥244,28.666 mg/d;.

[b] MD between quartile 4 and quartile 1 was calculated by ANCOVA.

[c] *P*-trend was determined using a test for linear trend across quartiles of BCAAs.

Model 1 was adjusted for age, sex.

Model 2 was additionally adjusted for BMI, smoking status, diabetes, hypertension, drinking status.

Model 3 was additionally adjusted for vitamin D, Fat, Carbohydrate.

Table S3. Covariate-adjusted mean changes in four physical performance indicators by quartiles of total BCAAs intakes in sup-population excluding those with myocardial infarction (n = 4273).

	Quartiles of BCAAs intake [a]									
	Q1		Q2		Q3		Q4		MD [b]	*P*-trend [c]
	Mean	SE	Mean	SE	Mean	SE	Mean	SE		
Handgrip strength										
Crude	21.4	0.26	22.8	0.26	23.6	0.26	24.4	0.26	2.97	<0.001
Model 1	22.6	0.22	23.6	0.22	24.2	0.22	24.3	0.22	1.68	<0.001
Model 2	22.7	0.29	23.6	0.28	24.2	0.29	24.4	0.28	1.66	<0.001
Model 3	22.6	0.29	23.5	0.28	24.1	0.29	24.4	0.28	1.76	<0.001
4-meter usual walking speed										
Crude	4.1	0.08	4.1	0.08	4.1	0.08	3.8	0.08	−0.31	0.018
Model 1	4.1	0.08	4.0	0.08	4.1	0.08	3.8	0.08	−0.28	0.051
Model 2	4.1	0.12	4.1	0.11	4.2	0.11	3.9	0.11	−0.23	0.145
Model 3	4.1	0.12	4.1	0.11	4.2	0.11	3.9	0.11	−0.23	0.138
4-meter fast walking speed										
Crude	3.1	0.03	3.0	0.03	2.9	0.03	2.8	0.03	−0.29	<0.001
Model 1	3.1	0.03	3.0	0.03	2.9	0.03	2.8	0.03	−0.26	<0.001
Model 2	3.1	0.04	3.0	0.04	2.9	0.04	2.8	0.04	−0.26	<0.001
Model 3	3.1	0.04	3.0	0.04	2.9	0.04	2.8	0.04	−0.26	<0.001
Repeated chair rises										
Crude	11.8	0.12	11.5	0.12	11.1	0.12	10.9	0.12	−0.87	<0.001
Model 1	11.7	0.12	11.5	0.12	11.1	0.12	11.0	0.12	−0.78	<0.001
Model 2	12.0	0.15	11.5	0.15	11.3	0.15	11.1	0.15	−0.84	<0.001
Model 3	12.0	0.15	11.5	0.15	11.3	0.15	11.1	0.15	−0.85	<0.001

Abbreviations: BCAAs, Branched-chain amino acids; Q, quartile; SE, standard error; MD, mean difference.

Note:

[a] Cutoff values of BCAA quartiles are as follows: Q1:<132,24.325 mg/d, Q2:132,24.325~182,97.085 mg/d, Q3:182,97.085~243,93.464 mg/d, Q4: ≥243,93.464 mg/d;

[b] MD between quartile 4 and quartile 1 was calculated by ANCOVA.

[c] *P*-trend was determined using a test for linear trend across quartiles of BCAAs.

Model 1 was adjusted for age, sex.

Model 2 was additionally adjusted for BMI, smoking status, diabetes, hypertension, drinking status.

Model 3 was additionally adjusted for vitamin D, Fat, Carbohydrate.

Table S4. Covariate-adjusted mean changes in four physical performance indicators by quartiles of total BCAAs intakes in sup-population excluding those with stroke (*n* = 4166).

	Quartiles of BCAAs intake [a]									
	Q1		Q2		Q3		Q4		MD [b]	*P*-trend [c]
	Mean	SE	Mean	SE	Mean	SE	Mean	SE		
Handgrip strength										
Crude	21.4	0.26	22.9	0.26	23.7	0.26	24.4	0.26	3.00	<0.001
Model 1	22.7	0.22	23.7	0.22	24.3	0.22	24.4	0.22	1.68	<0.001
Model 2	22.7	0.30	23.6	0.28	24.2	0.29	24.4	0.29	1.65	<0.001
Model 3	22.6	0.30	23.6	0.28	24.1	0.29	24.4	0.29	1.76	<0.001
4-meter usual walking speed										
Crude	4.1	0.08	4.0	0.08	3.9	0.08	3.9	0.08	−0.15	0.494
Model 1	4.1	0.08	4.0	0.08	3.9	0.08	4.0	0.08	−0.11	0.503
Model 2	4.1	0.12	4.1	0.11	4.0	0.12	4.1	0.12	−0.07	0.862
Model 3	4.1	0.12	4.1	0.11	4.0	0.12	4.1	0.12	−0.06	0.893
4-meter fast walking speed										
Crude	3.1	0.03	3.0	0.03	2.9	0.03	2.8	0.03	−0.27	<0.001
Model 1	3.1	0.03	3.0	0.03	2.9	0.03	2.8	0.03	−0.24	<0.001
Model 2	3.1	0.04	3.0	0.04	2.9	0.04	2.8	0.04	−0.25	<0.001
Model 3	3.1	0.04	3.0	0.04	2.9	0.04	2.8	0.04	−0.26	<0.001
Repeated chair rises										
Crude	11.7	0.12	11.4	0.12	11.0	0.12	10.8	0.12	−0.93	<0.001
Model 1	11.7	0.12	11.4	0.12	11.0	0.12	10.9	0.12	−0.83	<0.001
Model 2	11.9	0.15	11.2	0.15	11.2	0.15	11.0	0.15	−0.89	<0.001
Model 3	12.0	0.16	11.4	0.15	11.2	0.15	11.0	0.15	−0.91	<0.001

Abbreviations: BCAAs, Branched-chain amino acids; Q, quartile; SE, standard error; MD, mean difference.

Note:

[a] Cutoff values of BCAA quartiles are as follows: Q1:<131,83.665 mg/d, Q2:131,83.665~18271.014 mg/d, Q3:182,71.014~242,72.820 mg/d, Q4: ≥242,72.820 mg/d;

[b] MD between quartile 4 and quartile 1 was calculated by ANCOVA.

[c] *P*-trend was determined using a test for linear trend across quartiles of BCAAs.

Model 1 was adjusted for age, sex.

Model 2 was additionally adjusted for BMI, smoking status, diabetes, hypertension, drinking status.

Model 3 was additionally adjusted for vitamin D, Fat, Carbohydrate.

Table S5. Covariate-adjusted mean changes in four physical performance indicators by quartiles of total BCAAs intakes in sup-population excluding those with angina pectoris (*n* = 4256).

	Quartiles of BCAAs intake [a]									
	Q1		Q2		Q3		Q4		MD [b]	*P*-trend [c]
	Mean	SE	Mean	SE	Mean	SE	Mean	SE		
Handgrip strength										
Crude	21.4	0.26	22.8	0.26	23.6	0.26	24.4	0.26	3.03	<0.001
Model 1	22.6	0.22	23.6	0.22	24.2	0.22	24.3	0.22	1.70	<0.001
Model 2	22.7	0.29	23.5	0.28	24.1	0.29	24.4	0.28	1.69	<0.001
Model 3	22.6	0.29	23.5	0.28	24.1	0.29	24.3	0.28	1.77	<0.001
4-meter usual walking speed										
Crude	4.1	0.08	4.1	0.08	4.0	0.08	3.9	0.08	−0.15	0.484
Model 1	4.1	0.08	4.0	0.08	3.9	0.08	4.0	0.08	−0.12	0.517
Model 2	4.1	0.12	4.1	0.11	4.0	0.11	4.1	0.11	−0.08	0.867
Model 3	4.1	0.12	4.1	0.11	4.0	0.11	4.1	0.11	−0.08	0.889
4-meter fast walking speed										
Crude	3.1	0.03	3.0	0.03	2.9	0.03	2.8	0.03	−0.27	<0.001
Model 1	3.1	0.03	3.0	0.03	2.9	0.03	2.8	0.03	−0.24	<0.001
Model 2	3.1	0.04	3.0	0.04	2.9	0.04	2.8	0.04	−0.25	<0.001
Model 3	3.1	0.04	3.0	0.04	2.9	0.04	2.8	0.04	−0.26	<0.001
Repeated chair rises										
Crude	11.8	0.12	11.5	0.12	11.1	0.12	10.9	0.12	−0.90	<0.001
Model 1	11.8	0.12	11.5	0.12	11.0	0.12	10.9	0.12	−0.80	<0.001
Model 2	12.0	0.15	11.5	0.15	11.2	0.15	11.1	0.15	−0.86	<0.001
Model 3	12.0	0.15	11.5	0.15	11.2	0.15	11.1	0.15	−0.88	<0.001

Abbreviations: BCAAs, Branched-chain amino acids; Q, quartile; SE, standard error; MD, mean difference.

Note:

[a] Cutoff values of BCAA quartiles are as follows: Q1:<131,86.911 mg/d, Q2:131,86.911~182,59.170 mg/d, Q3:182,59.170~242,66.475 mg/d, Q4: ≥242,66.475 mg/d;

[b] MD between quartile 4 and quartile 1 was calculated by ANCOVA.

[c] *P*-trend was determined using a test for linear trend across quartiles of BCAAs.

Model 1 was adjusted for age, sex.

Model 2 was additionally adjusted for BMI, smoking status, diabetes, hypertension, drinking status.

Model 3 was additionally adjusted for vitamin D, Fat, Carbohydrate.
